# A novel five‐gene signature predicts overall survival of patients with hepatocellular carcinoma

**DOI:** 10.1002/cam4.3900

**Published:** 2021-05-02

**Authors:** Zhigang Wang, Leyu Pan, Deliang Guo, Xiaofeng Luo, Jie Tang, Weihua Yang, Yuxian Zhang, Anni Luo, Yang Gu, Yuxuan Pan

**Affiliations:** ^1^ Department of Hepatobiliary and Pancreas The First People's Hospital of Jingmen Jingmen China; ^2^ Department of Hepatobiliary and Pancreas Zhongnan Hospital of Wuhan University Wuhan China; ^3^ Department of Anesthesiology The First People's Hospital of Jingmen Jingmen China; ^4^ Department of Blood Transfusion The First People's Hospital of Jingmen Jingmen China

**Keywords:** Cox regression analysis, hepatocellular carcinoma, nomogram, prognosis, quantitative real‐time PCR

## Abstract

Hepatocellular carcinoma (HCC) is one of the most common public health challenges, worldwide. Because of molecular complexity and tumor heterogeneity, there are no effective predictive models for prognosis of HCC. This underlines the unmet need for accurate prognostic models for HCC. Analysis of GSE14520 data from gene omnibus (GEO) database identified multiple differentially expressed mRNAs (DEMs) between HCC and normal tissues. After randomly stratifying the patients into the training and testing groups, we performed univariate, lasso, and multivariable Cox regression analyses to delineate the prognostic gene signature in training set. We then used Kaplan–Meier plot, time‐dependent receiver operating characteristic (ROC), multivariable Cox regression analysis of clinical information, nomogram, and decision curve analysis (DCA) to evaluate the predictive and overall survival value of a novel five‐gene signature (*CNIH4*, *SOX4*, *SPP1*, *SORBS2*, and *CCL19*) within and across sets, separately and combined. We also validated the prognostic value of the five‐gene signature using The Cancer Genome Atlas—Liver Hepatocellular Carcinoma (TCGA‐LIHC), GSE54236 and International Cancer Genome Consortium (ICGC) sets. Multivariable Cox regression analysis revealed that the five‐gene signature and tumor node metastasis (TNM) stage were independent prognostic factors for overall survival of HCC patients in GSE14520 and TCGA‐LIHC. Combining TNM stage clinical pathological parameters and nomogram greatly improved the prognosis prediction of HCC. Further gene set enrichment analysis (GSEA) revealed enrichment of KEGG pathways related to cell cycle in the high‐risk group and histidine metabolism in the low‐risk group. Finally, all these five mRNAs are overexpressed between 12 pairs of HCC and adjacent normal tissues by quantitative real‐time PCR validation. In brief, a five‐gene prognostic signature and a nomogram were identified and constructed, respectively, and further validated for their HCC prognostic value. The five‐gene risk score together with TNM stage models could aid in rationalizing customized therapies in HCC patients.

## Graphical abstract

In brief, our study constructed and validated a five‐gene prognostic model and a nomogram that can predict the overall survival of patients with HCC. Our findings may guide the rationale of customized therapy in patients with varied TNM of HCC.

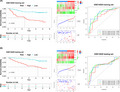

## 1 INTRODUCTION

Hepatocellular carcinoma (HCC) is the third leading cause of cancer‐related death, paralleling gastric cancer and only behind colorectal and lung cancer worldwide.^1^, ^2^, ^3^ The major risk factors for HCC include hepatitis B virus (HBV) and hepatitis C virus (HCV) infection, cirrhosis, and accumulation of aflatoxins in the liver.^4^ Since HCC is usually asymptomatic at early stages, treatment at the latter disease stage is often sub‐optimal. Although there has been a rapid development of HCC therapeutics, the ten year overall survival of HCC patients remains unsatisfactory.^5^, ^6^ Studies show that although conventional clinical parameters such as tumor node metastasis (TNM) stage, histologic grade and portal vein tumor thrombus (PVTT) could help predict HCC prognosis,^7^ they are limited by HCC heterogeneity. This underlines the urgent need to develop sensitive and reliable prognostic signatures, for optimal customized treatment.

Genome‐sequencing technology has revealed the prognostic power of gene signatures for HCC. Key molecular parameters for HCC prognosis include mRNA, lncRNA and microRNAs.^8^, ^9^, ^10^, ^11^ Analysis of publicly available genomic data has revealed multiple and efficient prognosis gene signatures. However, gene signatures alone while disregarding clinical parameters for predicting overall survival has been counterproductive. As such, it is important to combine novel gene signatures and clinical parameters for more efficient prognosis prediction.

In this study, we performed multiple bioinformatics analyses such as evaluation of differential expression mRNAs (DEMs), univariate Cox regression, least absolute shrinkage and selection operator (LASSO) Cox regression, multivariable Cox regression analysis, Kaplan–Meier plot, time‐dependent ROC, decision curve analysis (DCA), and gene set enrichment analysis (GSEA), to build and validate a model that combines a five‐gene signature and TNM stage, to predict the prognosis of HCC. Meanwhile, GSEA was performed to explore mechanisms underlying poor and better prognosis. Quantitative real‐time PCR was also performed to assess mRNA expression profiles in 12 pairs of HCC and adjacent normal tissues.

## 2 MATERIALS AND METHODS

### 2.1 Data source

The GSE14520 and GSE54236 two datasets contained HCC genes expression and clinical information were downloaded from GEO database in NCBI (https://www.ncbi.nlm.nih.gov/pmc/).^12^ We used the GPL3921 and GPL6480 to re‐annotate these probes, respectively. The method of normalization for these two datasets was used “normalizeBetweenArrays” function in “limma” package.^13^ Gene expression profile and clinical and survival information for HCC in The Cancer Genome Atlas—Liver Hepatocellular Carcinoma (TCGA‐LIHC) validation set was downloaded from UCSC Xena online website (http://xena.ucsc.edu/public/). The probe ID of TCGA‐LIHC expression was re‐annotated by GENCODE website (https://www.gencodegenes.org/). The method of normalization in TCGA‐LIHC was used log2 provided by UCSC Xena online website. The RNA‐seq data and clinical information of LIRI‐JP were downloaded from International Cancer Genome Consortium (ICGC) portal (https://daco.icgc.org/).

### 2.2 Differential expression mRNAs

Analysis of GSE14520 dataset for differently expressed mRNAs between HCC and non‐tumor tissues was performed using the “limma” package in R software.^13^ |log(FC)| > 1 and value of *p* < 0.05 were considered to be statistically significant.

### 2.3 Construction of prognostic gene signature

Samples from individuals with less than 1‐month overall survival time were excluded from subsequent analyses. Statistically significant differently expressed mRNAs (DEMs) were subjected to further univariate Cox regression analysis to evaluate their correlation with overall survival of HCC patients. Patients were randomly divided into training and testing sets by “caret” package in R software.^14^ LASSO regression analysis was then performed to evaluate the prognostic value of the DEMs.^15^ The predictive function of relevant prognostic mRNAs was derived after multivariable Cox regression. The risk score was expressed as: [(Coefficient_mRNA1_ * expression value of mRNA1) + (Coefficient_mRNA2_ * expression value of mRNA2) + (Coefficient_mRNA3_ * expression value of mRNA3) + ⋯ + (Coefficient_mRNAn_ * expression value of mRNAn)]. Patients were stratified into the high‐ and low‐risk survival groups based on optimum cut‐off risk scores. Factors impacting on survival between the two groups were analyzed based on the Kaplan–Meier survival curve and log‐rank test. Meanwhile, the predictive 1‐year, 3‐year, and 5‐year prognostic value of the gene signature was analyzed based on area under curve (AUC) values.^16^ Finally, the coefficients for all prognostic DEMs were used for testing and whole sets.

### 2.4 Independent validation of the prognostic gene signature and expression

The prognostic value of DEMs was validated using HCC data in TCGA‐LIHC, GSE54236, and ICGC dataset. The risk score for each patient was calculated using the previously described equation. The optional cut‐off value for the high‐ and low‐risk groups of patients was determined using the “surv_cutpoint” function in “survminer” package. Similarly, we used the Kaplan–Meier curve and time‐dependent ROC curve to evaluate the predictive value of DEMs signature in the validation dataset. Thereafter, the expression profile of DEMs between 50 pairs of tumors and normal adjacent tissues in TCGA‐LIHC was evaluated using paired *t*‐test. The value of *p* < 0.05 was considered statistically significant.

### 2.5 Independent prognostic value of the gene signature

To evaluate the prognostic value of DEMs, we performed univariate and multivariate analyses on all demographic and clinical parameters [gender, age, alanine transaminase (ALT), tumor size, multinodular, cirrhosis, serum alpha fetoprotein (AFP), and TNM stage] in GSE14520 dataset. Meanwhile, the same analysis was performed on TCGA‐LIHC data set [gender, age, body mass index (BMI), tumor grade, cancer status, and TNM stage]. The value of *p* < 0.05 was considered statistically significant.

### 2.6 Construction and validation of a nomogram model

Nomograms have been utilized in predicting the prognosis of multiple cancers.^17^, ^18^ Accordingly, it was utilized in predicting 1‐year, 3‐year, and 5‐year overall survival of HCC patients in the GSE14520 dataset, based on all independent prognostic factors identified in multivariate Cox analysis. At the same time, the predictive capacity of the nomogram was also evaluated using calibration curve and discrimination index. The concordance index (C‐index) for evaluating the discrimination of the nomogram was obtained by a bootstrap method, using 1000 resamples. The calibration curve was then plotted to visualize the predictive probability of the nomogram. Thereafter, we compared the performance of single and combined models using C‐index, time‐dependent ROC curve, and DCA packages in R software.^19^ Subsequently, we conducted the same operation in the independent validation set.

### 2.7 Gene set enrichment analysis

We performed gene set enrichment analysis (GSEA) of GSE14520 and TCGA‐LIHC sets using Kyoto Encyclopedia of Genes and Genomes (KEGG) pathway (https://www.gsea‐msigdb.org/gsea/index.jsp).^20^ Statistical significance was set at *p* < 0.05 and false discovery rate (FDR) *q* < 0.25.

### 2.8 Quantitative PCR for HCC and normal tissues

Quantitative PCR (qPCR) was performed on 12 pairs of HCC and corresponding adjacent tissues extracted from 12 different HCC patients. The pathological type of these tissues was validated at The First People`s Hospital of Jingmen, the department of pathology. qPCR was only performed on HCC and paired tissues of patients treated at our hospital as well as naive for neoadjuvant therapy before surgery. All participants consented to the study, and all protocols for this research were approved by the ethical committee of The First People`s Hospital of Jingmen, and conducted in accordance with.

Total RNA was extracted using TRIzol reagent (Invitrogen). Based on a NanoDrop spectrophotometer, the degree of UV absorbance at A260/A280 for ALL the RNAS was almost 2.0 (Thermo Scientific Inc.). cDNA was synthesized based on a PrimeScript RT reagent Kit with gDNAEraser (Takara), using the CFX Connect Real‐Time PCR Detection System (Bio‐Rad). We used SYBR Green (Toyobo), as the molecular probes, whereas *GAPDH* was used as the internal control. The amplicons were analyzed based on 2^−ΔΔCt^ equation. All primers were designed at the PrimerBank website (https://pga.mgh.harvard.edu/primerbank/). The sequences and Tm values of all primers were listed in Supporting Information Table S1.

### 2.9 Statistical analysis

Statistical analyses were performed using R (version 3.6.3). Categorical variables were analyzed using the Pearson chi‐squared or Fisher's exact test; with paired tissues analyzed using paired *t*‐test. Unpaired non‐normally distributed samples were analyzed using Wilcoxon test. The value of *p* < 0.05 was considered statistically significant.

## 3 RESULTS

### 3.1 DEMs analysis

**FIGURE 1:**
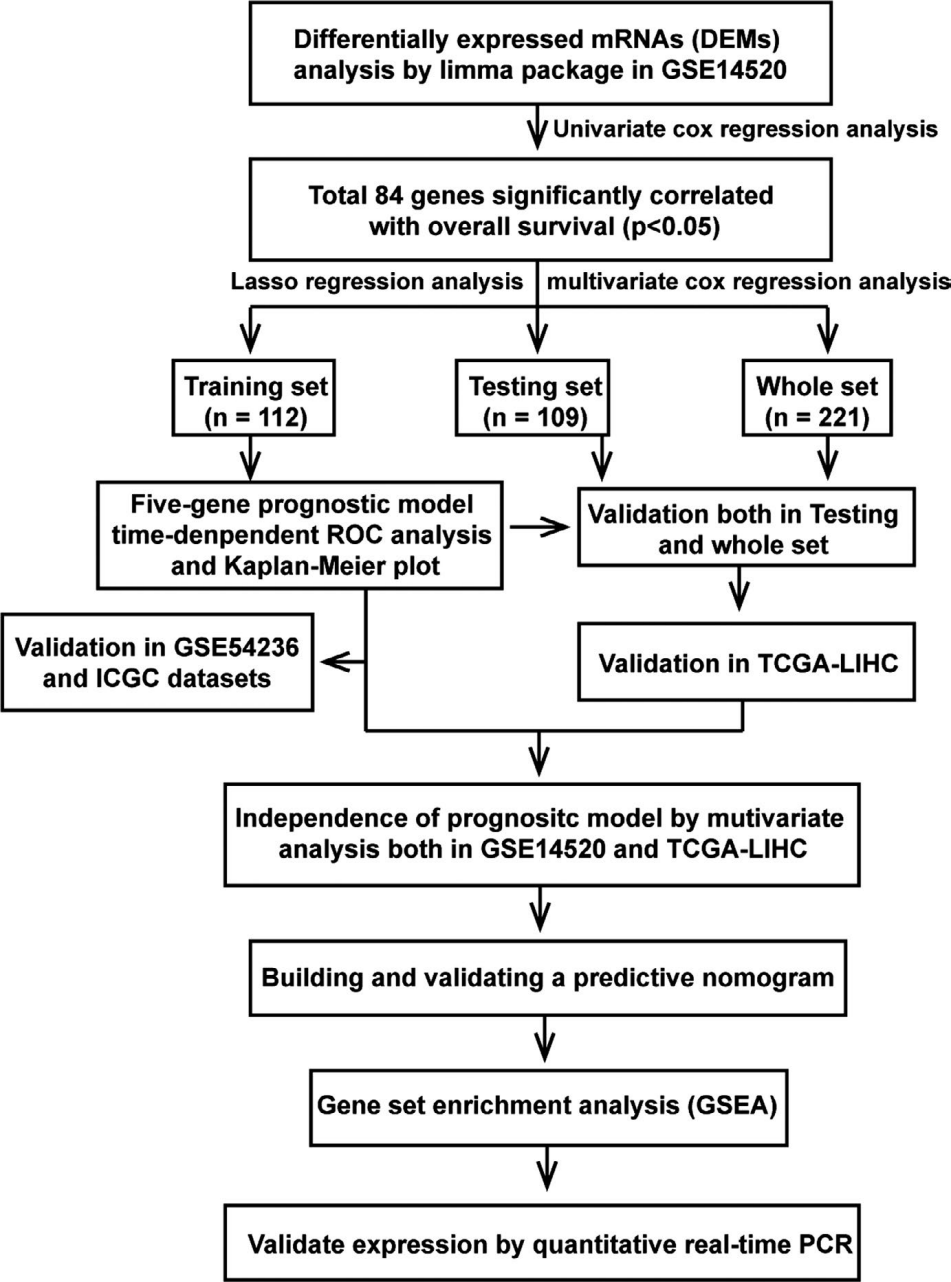
Flowchart of study design

An overview of the research design is show in Figure 1. After normalization of expression matrix, principal components analysis (PCA) analysis revealed a clear distinction between normal and tumor tissues (Supporting Information Figure S1A). In general, we identified 443 DEMs in 225 HCC and 220 normal tissues, in which 110 were upregulated, whereas 333 were downregulated. The heatmap is presented in Supporting Information Figure S1B, whereas the volcano plot is shown in Supporting Information Figure S1C.

### 3.2 Construction of the five‐gene prognostic gene signature

**FIGURE 2:**
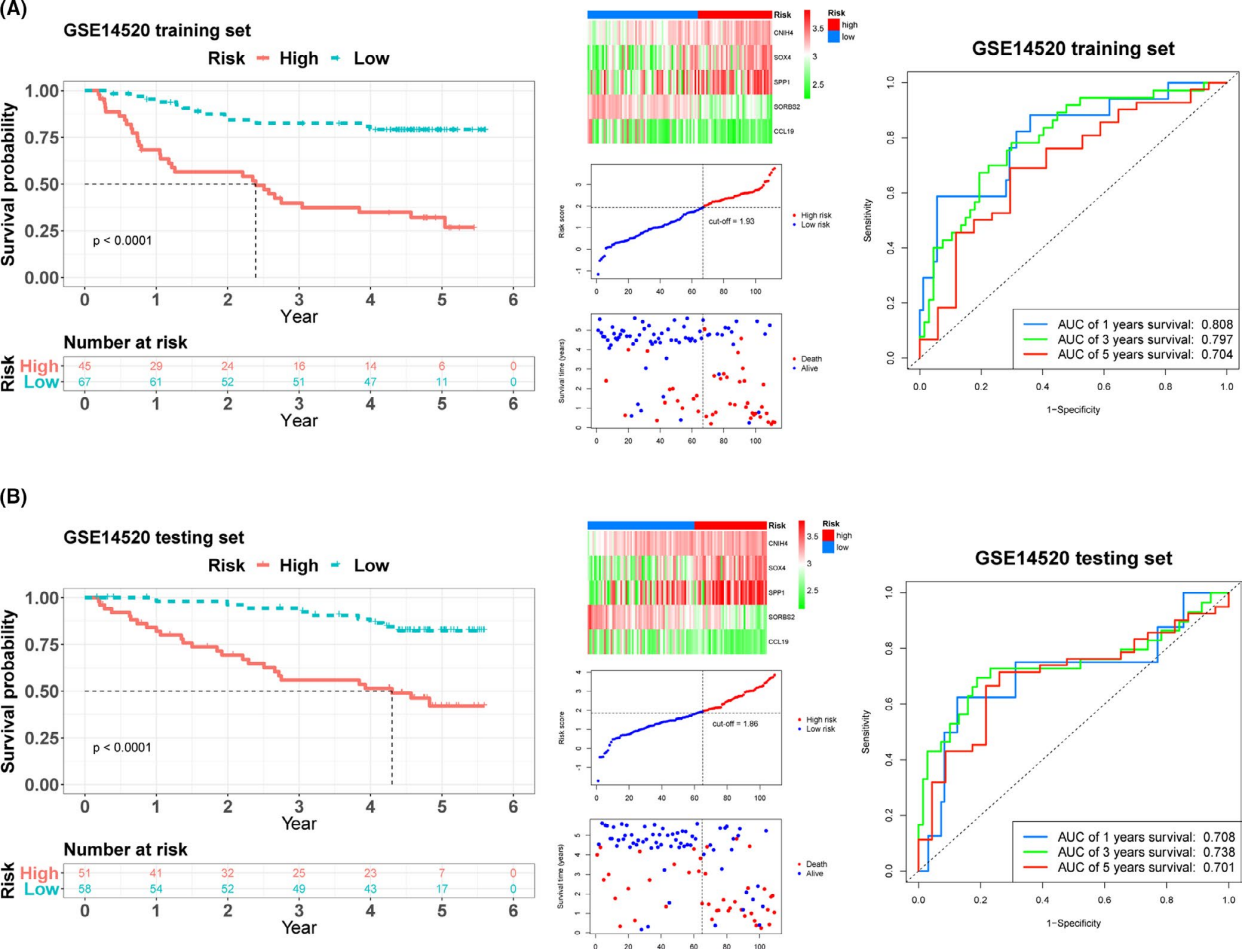
Kaplan–Meier survival analysis, risk score analysis, and time‐dependent receiver operating characteristic (ROC) analysis for the five‐gene signature in hepatocellular carcinoma (HCC). The Kaplan–Meier plot, five mRNAs heatmap, cut‐off value, survival states of patients, and time‐dependent ROC analysis in (A) training and (B) testing sets of GSE14520

**FIGURE 3:**
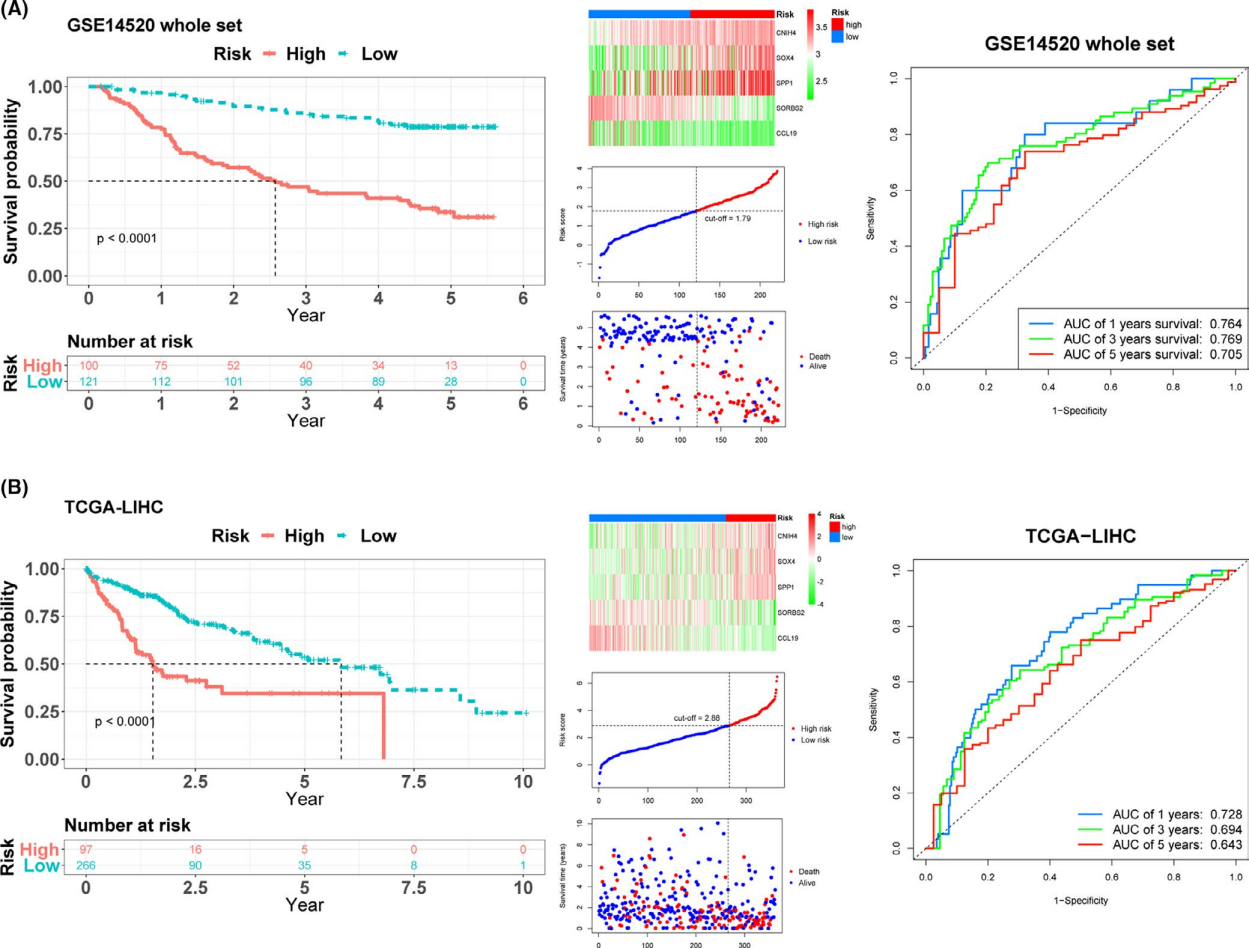
Kaplan–Meier survival analysis, risk score analysis and time‐dependent ROC analysis in whole and The Cancer Genome Atlas Liver Hepatocellular Carcinoma (TCGA‐LIHC) sets for the five‐gene signature in HCC. The Kaplan–Meier plot, five mRNAs heatmap, cut‐off value, survival states of patients, and time‐dependent ROC analysis in (A) whole and (B) TCGA‐LIHC sets

We constructed a new matrix incorporating all DEMs and overall survival data for HCC patients. In general, 221 patients with overall survival longer than (one month) were included. These patients were randomly classified into training set (*n* = 112) or testing set (*n* = 109). The characteristics for the two groups are shown in Supporting Information File S1. Univariate Cox regression analysis first revealed 84 mRNAs that correlated with overall survival of HCC patients. Thereafter, LASSO analysis identified and quantified the significance of seven DEMs in the training set (Supporting Information File S2). We chose 1000 iterations for selecting the optimal lambda parameters and corresponding coefficients in LASSO Cox regression. Further multivariable Cox regression analysis of the seven mRNAs found only five key mRNAs, including those for cornichon family AMPA receptor auxiliary protein 4 (*CNIH4*), SRY‐box transcription factor 4 (*SOX4*), secreted phosphoprotein 1 (*SPP1*), sorbin and SH3 domain containing 2 (*SORBS2*), and C‐C motif chemokine ligand 19 (*CCL19*) to be the most significant mRNAs associated with survival of HCC patients. The prognostic risk score was derived as follows. Patients were stratified into the high‐ (*n* = 45) and low‐risk groups (*n* = 67) based on individual risk scores. Time‐dependent ROC and Kaplan–Meier curves evaluated the overall predictive potential of the five gene sets for overall survival of patients in the training set. Similar analysis was performed on testing set as well as pooled set. AUCs for 1‐year, 3‐year, and 5‐year overall survival in three sets [(0.70) (Figures 2A,B and 3A)], revealed the strength of the five‐gene signature in predicting overall survival.

### 3.3 External validation of the prognostic gene signature

The risk scores for data in TCGA‐LIHC dataset were calculated as described in the preceding section. After setting the optimal cut‐off value (2.88), the patients were also divided into the high‐risk (*n* = 97) and low‐risk (*n* = 266) groups. The Kaplan–Meier plot showed the same result with three sets. The AUCs for 1‐year, 3‐year, and 5‐year overall survival were 0.728, 0.694, and 0.643, respectively (Figure 3B), validating the survival predictive value of the five‐gene signature in HCC. On the other hand, GSE54236 dataset was also used for validation. All patients were separated into the high‐risk (*n* = 33) and low‐risk (*n* = 48) groups by choosing optimal cut‐off value (2.43). Patients in high‐risk group had less survival time compared with those in low‐risk group (Supporting Information Figure S2A). Meanwhile, the AUCs for 1‐year, 3‐year, and 5‐year overall survival in GSE54236 were 0.735, 0.633, and 0.797, respectively (Supporting Information Figure S2B). The final validation set ICGC, 232 patients were divided into the high‐risk (*n* = 28) and low‐risk (*n* = 184) groups after choosing optimal cut‐off value (2.31). The survival result had the same tendency with below (Supporting Information Figure S2C). In addition, the AUCs for 1‐year, 3‐year, and 5‐year overall survival in ICGC validation cohort were 0.688, 0.714, and 0.717 (Supporting Information Figure S2D).

### 3.4 Independent prognostic role of the gene signature

**FIGURE 4:**
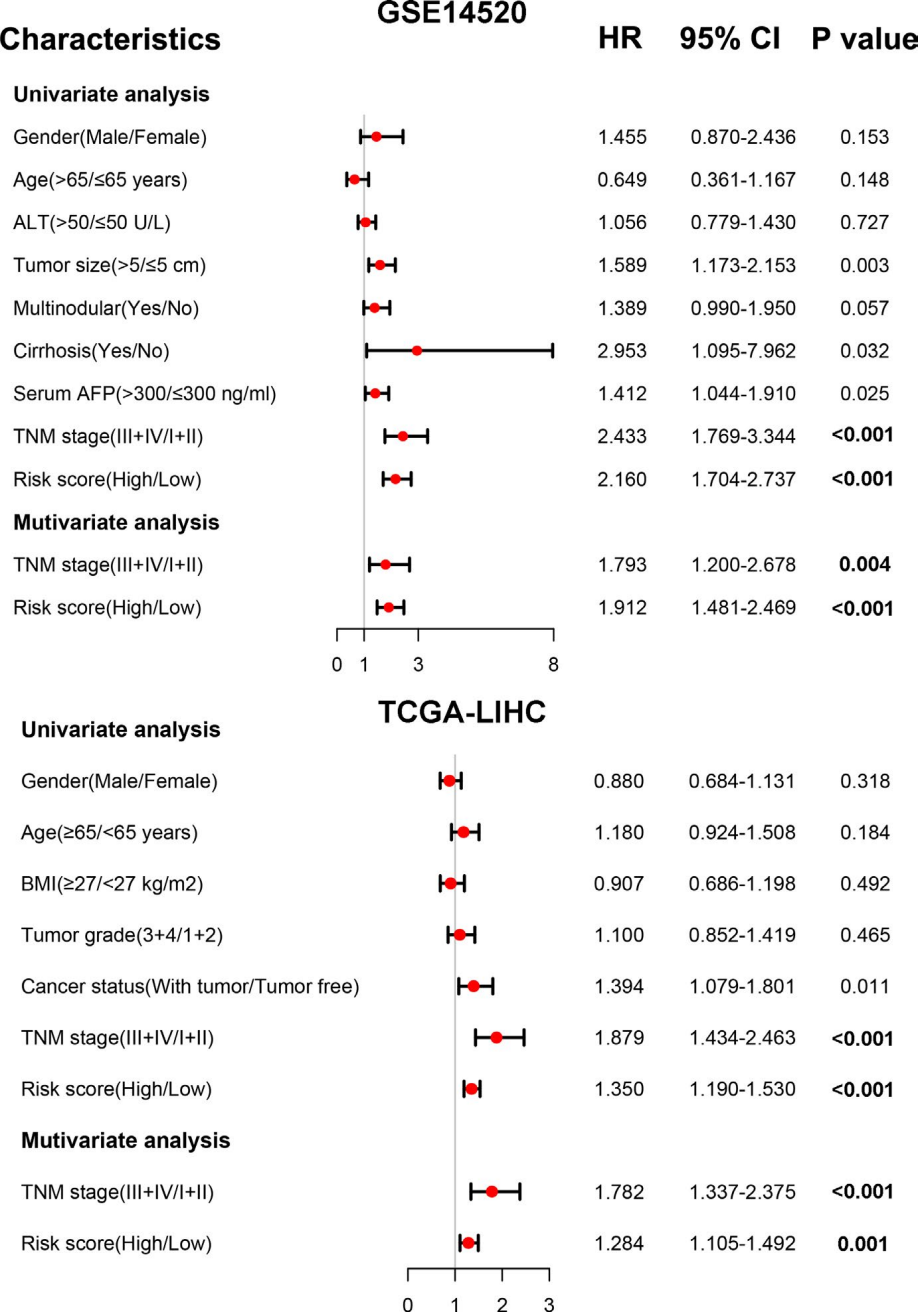
Forest plot of the univariate and multivariate Cox regression analysis in two sets. A value of *p* < 0.05 was considered significant. ALT, alanine transaminase; AFP, alpha fetoprotein; BMI, body mass index; TNM, tumor node metastasis

**FIGURE 5:**
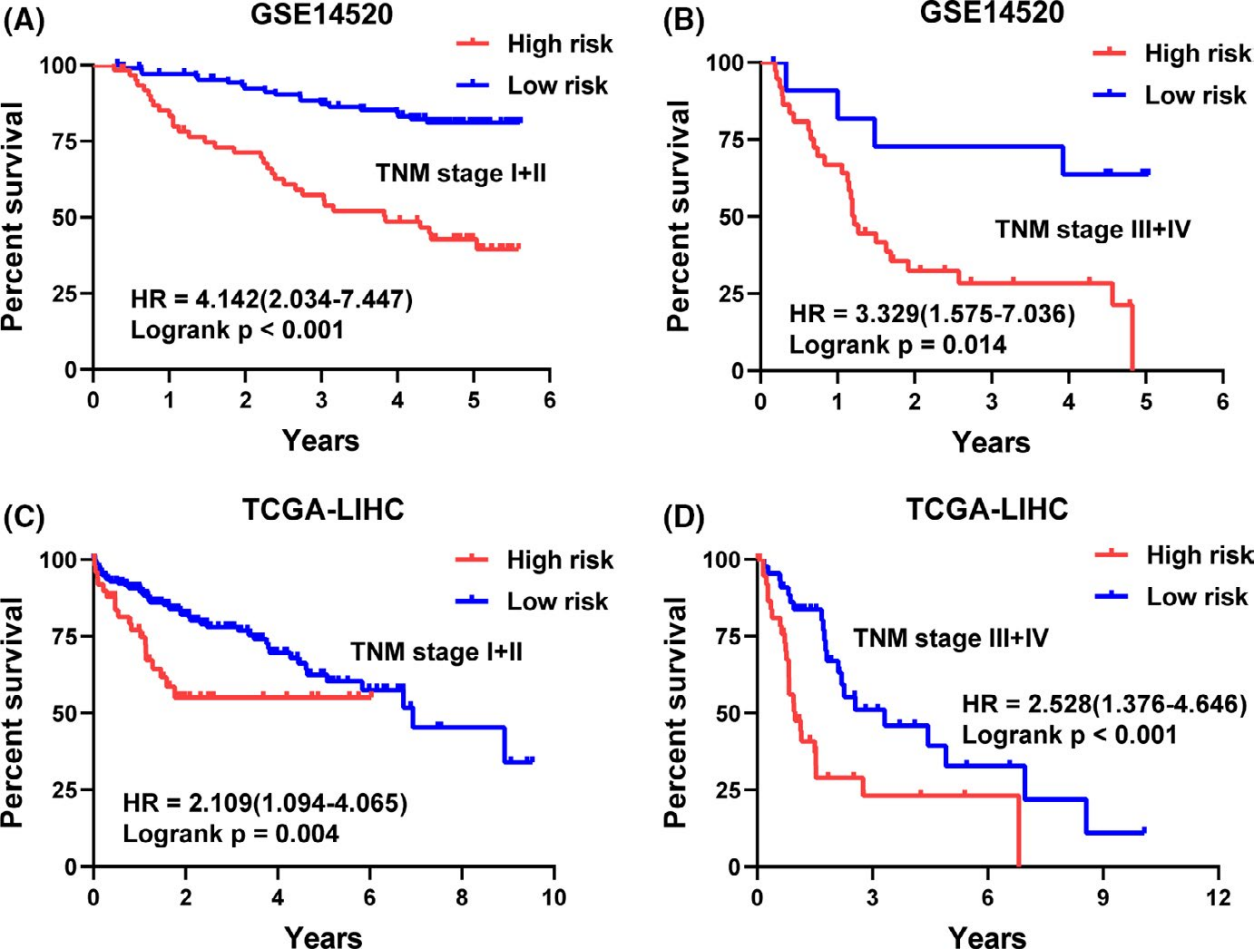
Kaplan–Meier plots of the five‐gene signature in different TNM stage of HCC patients. Patients in the high‐risk group showed poorer overall survival compared with those in the low‐risk group in (A, C) TNM stage I+II and (B, D) TNM stage III + IV in GSE14520, and TCGA‐LIHC sets

After constructing the HCC predictive model for the five‐gene signature for the high‐ and low‐risk groups, we created a table for clinical information patients in GSE14520 dataset, including gender, age, ALT, tumor size, multinodular, cirrhosis, serum AFP, and TNM stage. Missing information was labeled as “Not Available.” Analysis of clinical data revealed higher risk score was strong and positive correlation with advanced age, larger tumor size, cirrhosis, higher AFP, and advanced TNM stage. Meanwhile, TCGA‐LIHC clinical data on its part revealed a strong positive correlation between higher risk score and both advanced TNM stage and histologic grade (Table 1). The significance of the five‐gene signature was validated by univariate and multivariate Cox regression analysis of data in the GSE14520 and TCGA‐LIHC datasets, in which both TNM stage and risk prognostic model were found to be independent prognostic factors for overall survival of patients with HCC (Figure 4). Also, patients in the high‐risk group exhibited poorer overall survival compared with those in the low‐risk group, both for low (I + II) and high TNM stage (III + IV; Figure 5A–D).

**TABLE1 1:** The clinical information of five‐gene signature in two datasets

Characteristics	GSE14520			TCGA‐LIHC		
	High risk	Low risk	*p* value	High risk	Low risk	*p* value
Gender			0.534			0.698
Female	12	18		30	88	
Male	88	103		67	178	
Age (years)			0.023			0.511
≤65	94	102		55	161	
>65	6	19		42	105	
ALT (U/L)			0.11			
≤50	53	77				
>50	47	44				
Tumor size (cm)			0.003			
≤5	53	87				
>5	47	33				
Multinodular			0.058			
No	74	102				
Yes	26	19				
Cirrhosis			0.041			
No	4	14				
Yes	96	107				
Serum AFP (ng/ml)			<0.001			
≤300	41	77				
>300	59	41				
TNM stage			<0.001			<0.001
I + II	62	108		53	201	
III + IV	37	12		35	42	
BMI (kg/m^2^)						0.097
<27				66	154	
≥27				24	88	
Tumor grade						0.004
1 + 2				50	180	
3 + 4				46	82	
Cancer status						0.535
Tumor free				58	173	
With tumor				30	76	

Note

*p* < 0.05 was considered significant.

Abbreviations: AFP, alpha fetoprotein; ALT, alanine transaminase; BMI, body mass index; TNM, tumor node metastasis.

### 3.5 Construction and validation of a predictive nomogram

**FIGURE 6:**
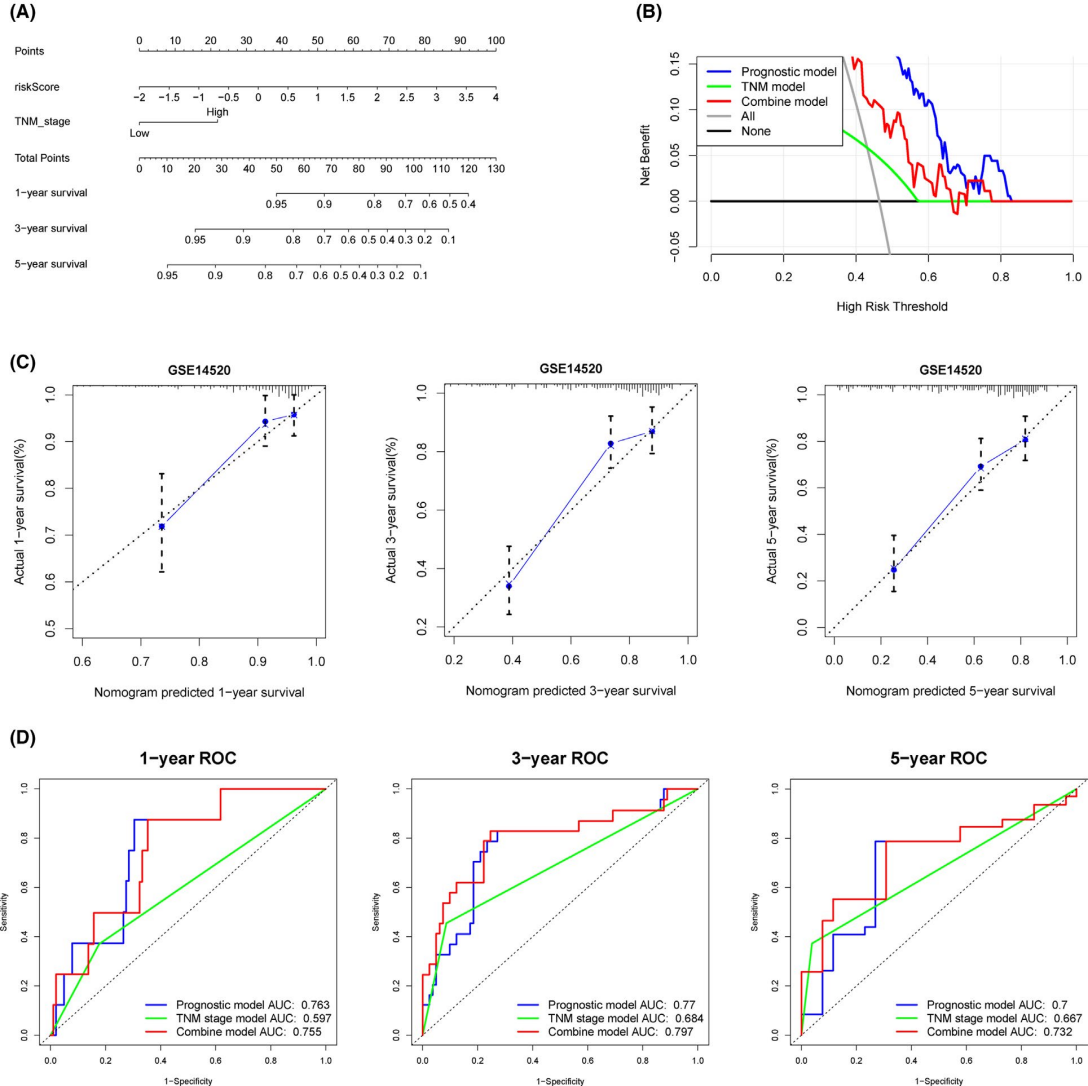
A predictive nomogram building and validating in GSE14520 set. A, The nomogram was built by two independent prognostic factors. B, The decision curve analysis (DCA) of prognostic, TNM stage, and combined models for 5‐year overall survival. C, The calibration plots for internal validation of the nomogram for 1‐year, 3‐year, and 5‐year survival, respectively. D, The time‐dependent ROC curves of the nomograms compared for 1‐year, 3‐year, and 5‐year overall survival, respectively

We used TNM stage data in GSE14520 to construct a prognostic nomogram (Figure 6A). Based on the calibration analysis of 1‐year, 3‐year, and 5‐year survival prediction, the nomogram best predicted the 5‐year overall survival rate of patients (Figure 6C). The C‐index values for prognostic model, TNM stage model and a combination of models were 0.716, 0.624, and 0.737, respectively. The ROCs for prognostic, TNM stage and combined models were also analyzed by “TimeROC” package^21^ (Figure 6D). The decision curve analysis (DCA) revealed that compared with prognostic and TNM models, the hybrid model was more superior in predicting 5‐year overall survival rate of patients (Figure 6B).

**FIGURE 7:**
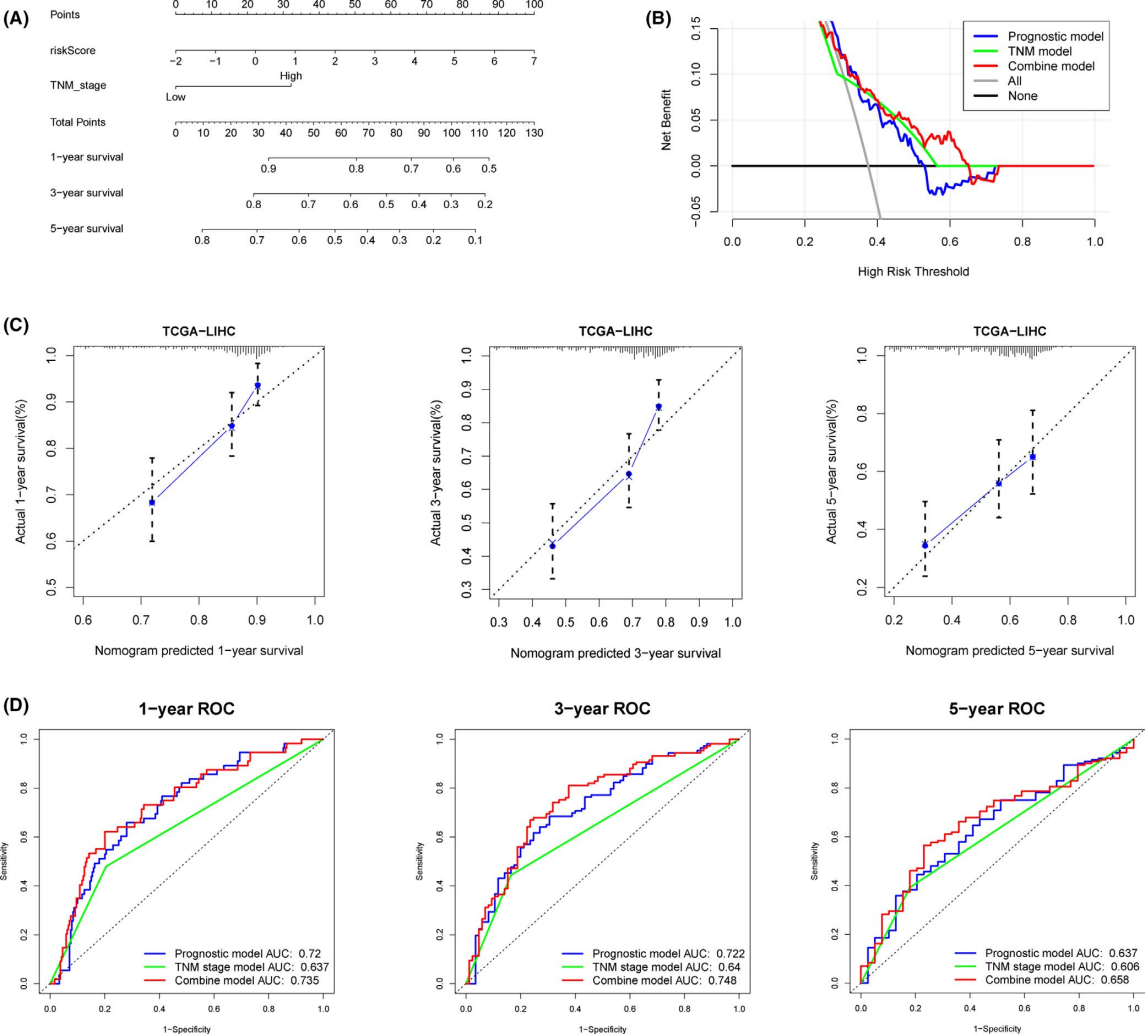
A predictive nomogram building and validating in TCGA‐LIHC set. A, The nomogram was built based on two independent prognostic factors. B, The decision curve analysis (DCA) of prognostic, TNM stage, and combined models for 5‐year overall survival. C, The calibration plots for internal validation of the nomogram for 1‐year, 3‐year, and 5‐year survival, respectively. D, The time‐dependent ROC curves of the nomograms compared for 1‐year, 3‐year, and 5‐year overall survival, respectively

Nomogram for two independent prognostic factors identified in TCGA‐LIHC dataset was also constructed (Figure 7A). The calibration plots for 1‐year, 3‐year, and 5‐year also revealed that the nomogram best predicted a 5‐year overall survival of HCC patients (Figure 7C). The C‐index values for prognostic, TNM stage and hybrid model were 0.671, 0.590, and 0.667, respectively. In addition, the AUCs for 1‐year, 3‐year, and 5‐year were all greater than 0.65 (Figure 7D). Superior overall 5‐year survival prediction was realized after constructing a hybrid model, featuring the prognostic and TNM model (Figure 7B).

Taken together, combining prognostic and TNM stage model confers excellent predictive potential for overall survival for HCC patients.

### 3.6 Mutation characteristics and gene set enrichment analysis

**FIGURE 8:**
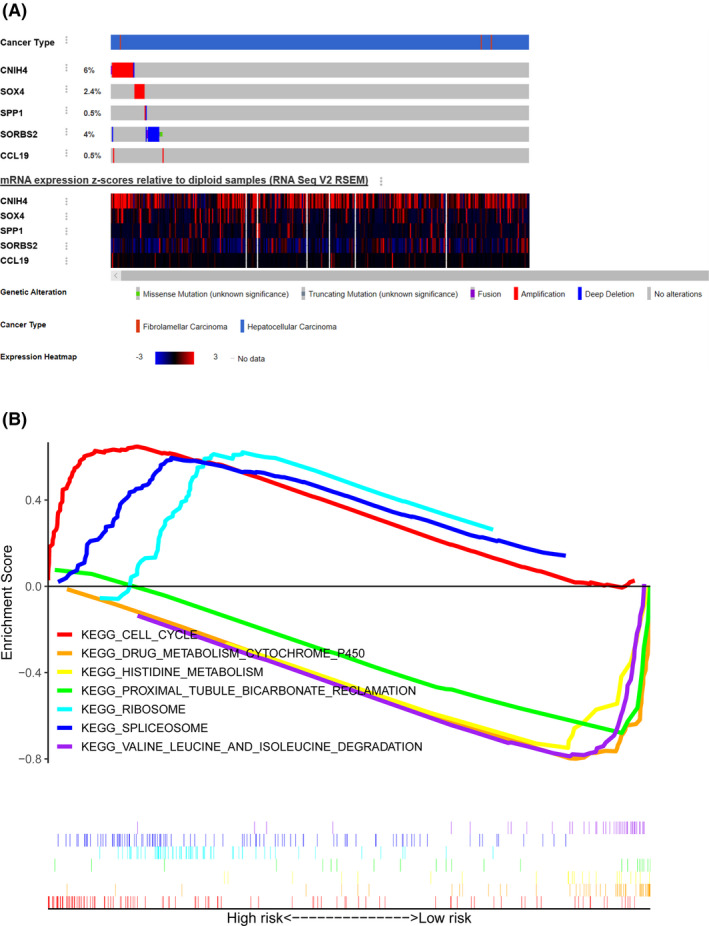
Mutation information and gene set enrichment analysis (GSEA). A, The mutation information of five prognostic genes in cBioPortal online website. B, Seven obvious Kyoto Encyclopedia of Genes and Genomes (KEGG) pathways enriched in the high‐ and low‐risk groups in GSE14520 set

To find the deeper value for the five‐gene signature, we searched through cBioPortal online website to explore the mutation blueprints of each gene. We found *CNIH4* to be overexpressed in 6% of the samples, and 4% deep deletion in *SORBS2*, but the other genes exhibited no apparent changes (Figure 8A). KEGG analysis revealed 37 enriched pathways in GSE14520. In particular, spliceosome, ribosome, cell cycle, and basal transcription factors were enriched in the high‐risk group, whereas multiple metabolic pathways such as histidine, tyrosine, butanoate, and fatty acid metabolism were enriched in the low‐risk group (Figure 8B, Supporting Information File S3).

### 3.7 External validation in expression

We validated the expression pattern of the novel gene signature in TCGA‐LIHC dataset using 50 pairs of normal and HCC tissues. Expect for *SORBS2*, all of the other four genes were differentially expressed between tumor and normal tissues, (Supporting Information Figure S3A). Risk scores were also significantly different between low and high TNM stage, both in GSE14520 and TCGA‐LIHC datasets. Furthermore, risk score for low and high histologic grade were also significantly different between the two groups (Supporting Information Figure S3B).

qPCR further revealed significant differential mRNA expression for the five novel genes between 12 pairs of HCC and normal tissues (Supporting Information Figure S3C). In general, an aberrant expression profile for a five‐gene signature was demonstrated in HCC.

## 4 DISCUSSION

Hepatocellular carcinoma causes significant mortalities worldwide.^22^ Due to its complex pathogenesis, it is extremely difficult to develop satisfactory predictive model for overall survival of HCC patients. Current models rely on tradition clinical indicators such as TNM stage, histologic grade, and portal vein tumor thrombus (PVTT), which have been shown to be semi‐efficient. Moreover, HCC heterogeneity has cemented greater need to identify novel prognostic biomarkers for more precise prognostic models. Compared with models based on single factors, hybrid prognostic models could enhance prognosis predictive efficacy.

Analysis of GSE14520 validated by TCGA‐LIHC, GSE54236, and ICGC cohort datasets revealed a novel five‐gene signature (*CNIH4*, *SOX4*, *SPP1*, *SORBS2*, and *CCL19*) for more accurate prognosis prediction of HCC. Univariate and multivariate analysis of clinical data in GSE14520 and TCGA‐LIHC two datasets revealed TNM stage and the five‐gene signature to be two independent prognostic factors for overall survival of HCC patients. Meanwhile, compared with the low‐risk group, patients in the high‐risk group had significant poorer overall survival for all TNM stages (I–IV). A nomogram combining the five‐gene signature and TNM stage models greatly improved the prognostic prediction of HC, in particular by delineating HCC patients more accurately customized treatment. A study reported that a novel nomogram for predicting survival of HCC patients was constructed by autophagy‐related genes.^23^ This model combined risk group, tumor size, and cirrhosis three parameters. Compared with this model, our model could predict short time survival time of HCC patients and had higher AUCs for 1‐year, 3‐year, and 5‐year, which means our five‐gene signature combined TNM stage could have better advantages. Zhou et al has constructed a 10‐gene signature which has very high AUCs for predicting 1‐year, 3‐year, and 5‐year of HCC patients both in training, testing, and independent sets.^24^ However, the calibration plots of 5‐year for nomogram performed relatively poorer compared with ours, which mean our model was more accurate for predicting 5‐year survival of HCC patients.

Taken together, these findings underline the role of a five‐gene signature model in predicting the overall survival of patients with HCC. GSEA analysis revealed several KEGG pathways for the five‐gene signature such as cell cycle and multiple metabolic pathways were significantly enriched, which showed the HCC patients those in the high‐risk group might activate HCC cell cycle to influence survival time. And those in the low‐risk group had longer survival time because of inhibition of metabolism cytochrome P450, histidine metabolism, and so on. The results of GSEA analysis might unravel the underlying molecular mechanisms for poor or better survival. Analysis of 50 pairs of normal and HCC tissues in the TCGA‐LIHC dataset revealed that except for *SORBS2*, the four genes were differentially expressed between tumor and normal tissues. Accordingly, we used 12 pairs of normal and HCC tissues obtained at our hospital to validate the above expression profile.

Cornichon family AMPA receptor auxiliary protein 4 (*CNIH4*) participates in regulation of G protein‐coupled receptors (GPCRs), involved in transporting proteins from the endoplasmic reticulum (ER) to the functional site (cell surface). Differential expression of *CNIH4* resulted in the retention of GPCRs.^25^ *CNIH4* gene, which encodes a member of CORNICHON family, an evolutionarily conserved TGFα exporter, is essential for metastasis of colon cancer cells. At the same time, *CNIH4* is regulated by *TMED9* activity.^26^ However, the role of *CNIH4* in HCC remains unclear. *SOX4* is a member of a highly conserved transcription factor SOX (SRY‐Box) family, with a typical DNA‐binding HMG domain.^27^ Studies show that the overexpression of *SOX4* promotes metastasis of HCC. Through immunoprecipitation and gene ablation, two *SOX4* target genes that influence HCC metastasis have been identified and validated.^28^ One study demonstrated that HMG box domain of *SOX4* interacts and inhibits p53‐mediated transcription of Bax. More importantly, *SOX4* overexpression strongly inhibits p53‐induced Bax expression and subsequent repression of p53‐mediated apoptosis induced by gamma irradiation of HCC.^29^ *SPP1* is an arginine‐glycine‐aspartate (RGD), containing multiple phosphoproteins. It participates in tumor metastasis, mediated by directly stimulated and migration of macrophages.^30^ Genetic polymorphisms of SPP1 gene are associated with HBV clearance and onset age of HCC, underlying the molecular mechanisms in HBV clearance and HCC progression.^31^ *SORBS2* is essential in regulating cell adhesion and actin/cytoskeletal organization. A recent study reported *SORBS2* could suppress metastatic colonization in ovarian cancer through multiple mechanisms.^32^ In a separate study, *SORBS2* expression was found to be downregulated in HCC, a phenomenon associated with metastasis, TNM stage, and prognosis of HCC. Mechanistically, *SORBS2* participates in the suppression of HCC tumourigenesis and metastasis via post‐transcriptional modulation of *RORA* expression, in particular by binding on its mRNA.^33^ A separate study implicated *MEF2D* for *SORBS2* downregulation and inhibition of HCC metastasis through the c‐Abl /ERK signaling pathway. This regulatory cascade is a potential prognostic marker or therapeutic target for HCC.^34^ *CCL19* not only participate in inflammatory and immunological responses, but also modulates recirculation and homing of lymphocytes. A separate but related study showed knock‐down of CC chemokine receptor like 1 (*CCRL1*) inhibits the expression of *CCL19* and *CCL21*. By targeting *CCL19* and *CCL21*, *CCRL1* modulates CCR7 binding by these molecules. This in turn minimizes harmful effects of CCR7, including undesirable activation of Akt‐GSK3 pathway in tumor cells.^35^ Nonetheless, the precise roles of *CCL19* in HCC are not well understood.

In general, we identified and validated a novel five‐gene signature prognostic model by plenty datasets and constructed a nomogram for predicting overall survival of HCC patients. Combining TNM stage and clinical pathological parameters greatly improved the predictive potential, particularly the 5‐year overall survival of HCC patients. qPCR validated the significant differential expression of these five genes between tumor and normal tissues. Our findings notwithstanding, this study suffered several limitations. First, the external validation datasets were still needed to increase. Second, this study did not explore the expression and prognostic effects of the five genes at the protein level. Third, the reliability of score model needs further clinical validation. As such additional clinical studies are necessary to validate findings of this study.

## 5 CONCLUSION

In brief, our study constructed and validated a relatively stable five‐gene prognostic model and a nomogram that could predict the overall survival of patients with HCC. Our findings may guide the rationale of customized therapy in patients with varied TNM of HCC.

## COMPETING INTERESTS

There was no conflict of interests.

## CONSENT FOR PUBLICATION

Not applicable.

## AUTHOR CONTRIBUTIONS

Yang Gu, Yuxuan Pan, Zhigang Wang, and Leyu Pan designed this study and analyzed the data. Deliang Guo performed quantitative real‐time PCR. Xiaofeng Luo, Jie Tang, Weihua Yang, Yuxian Zhang, and Anni Luo wrote the manuscript. All authors reviewed the manuscript.

## Supporting information

Fig S1

Fig S2

Fig S3

Table S1

## Data Availability

The source of all data during this research is included in this article. The R codes used in this study are available from the corresponding author by request.
