# Supplementary figures and images for: A novel five‐gene signature predicts overall survival of patients with hepatocellular carcinoma
Source: Cancer Med. 2021 May 2;10(11):3808–21. doi: 10.1002/cam4.3900 (PMC8178492; doi:10.1002/cam4.3900)

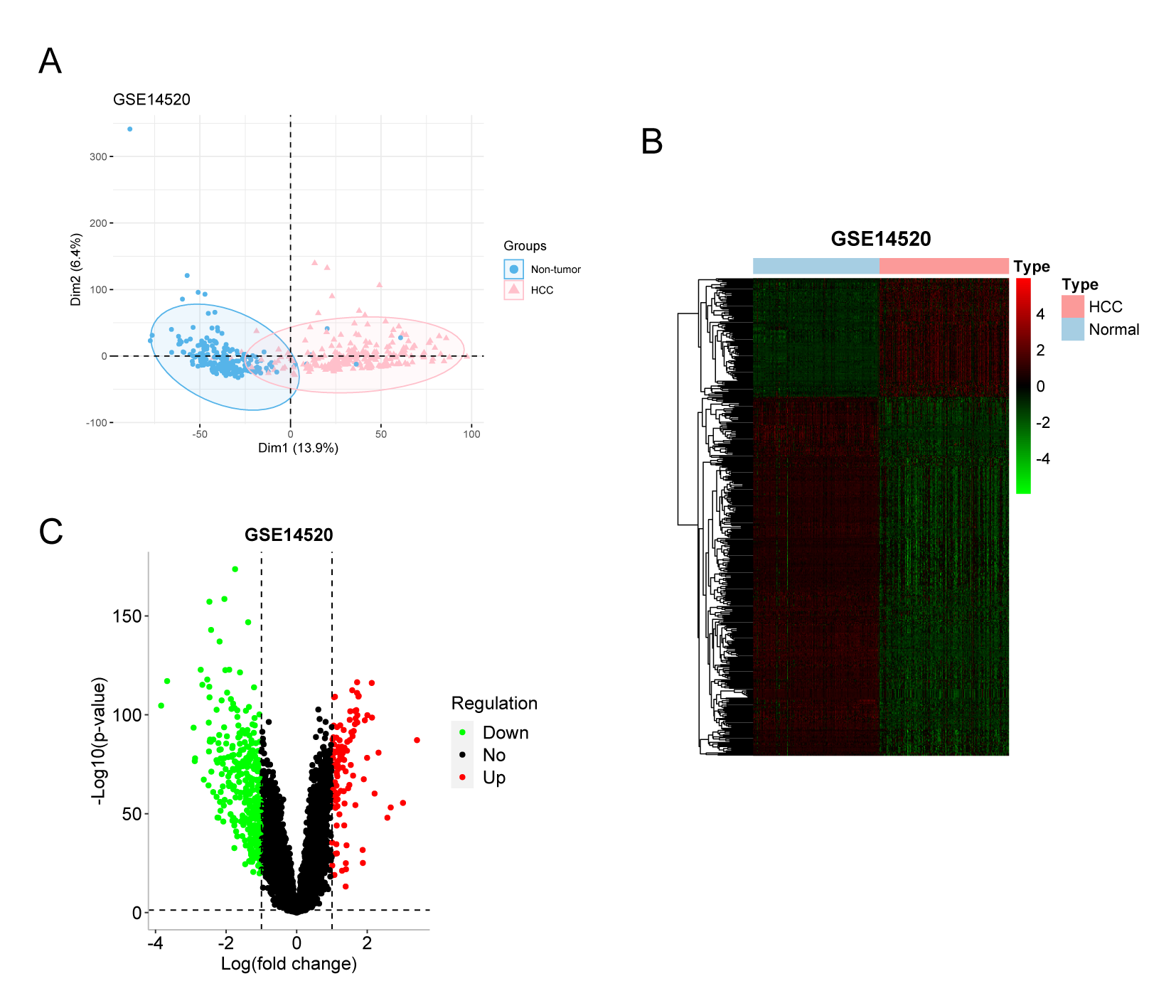

Supplement: Supplementary file 1 — Fig S1 [file CAM4-10-3808-s008.tif]

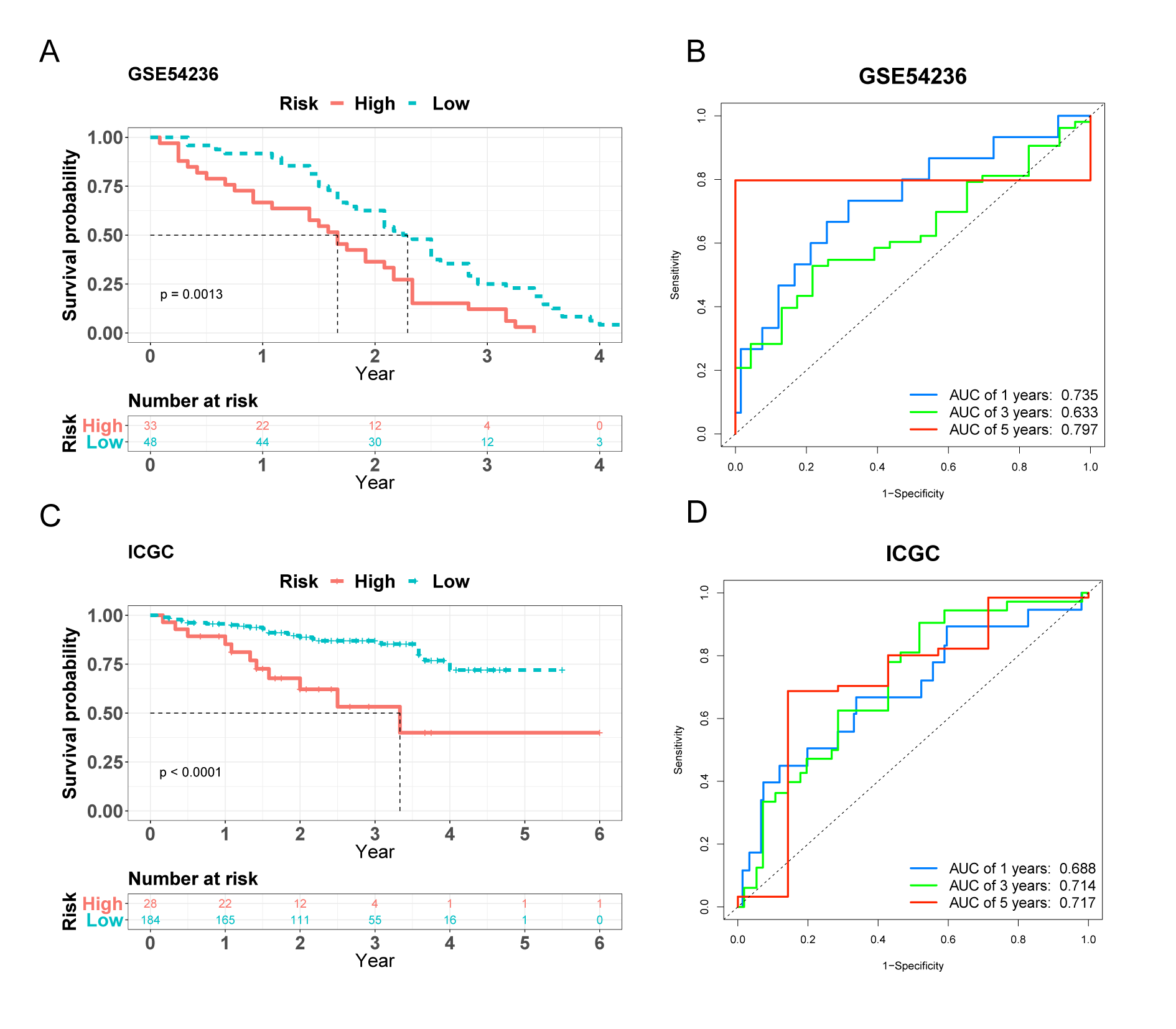

Supplement: Supplementary file 2 — Fig S2 [file CAM4-10-3808-s002.tif]

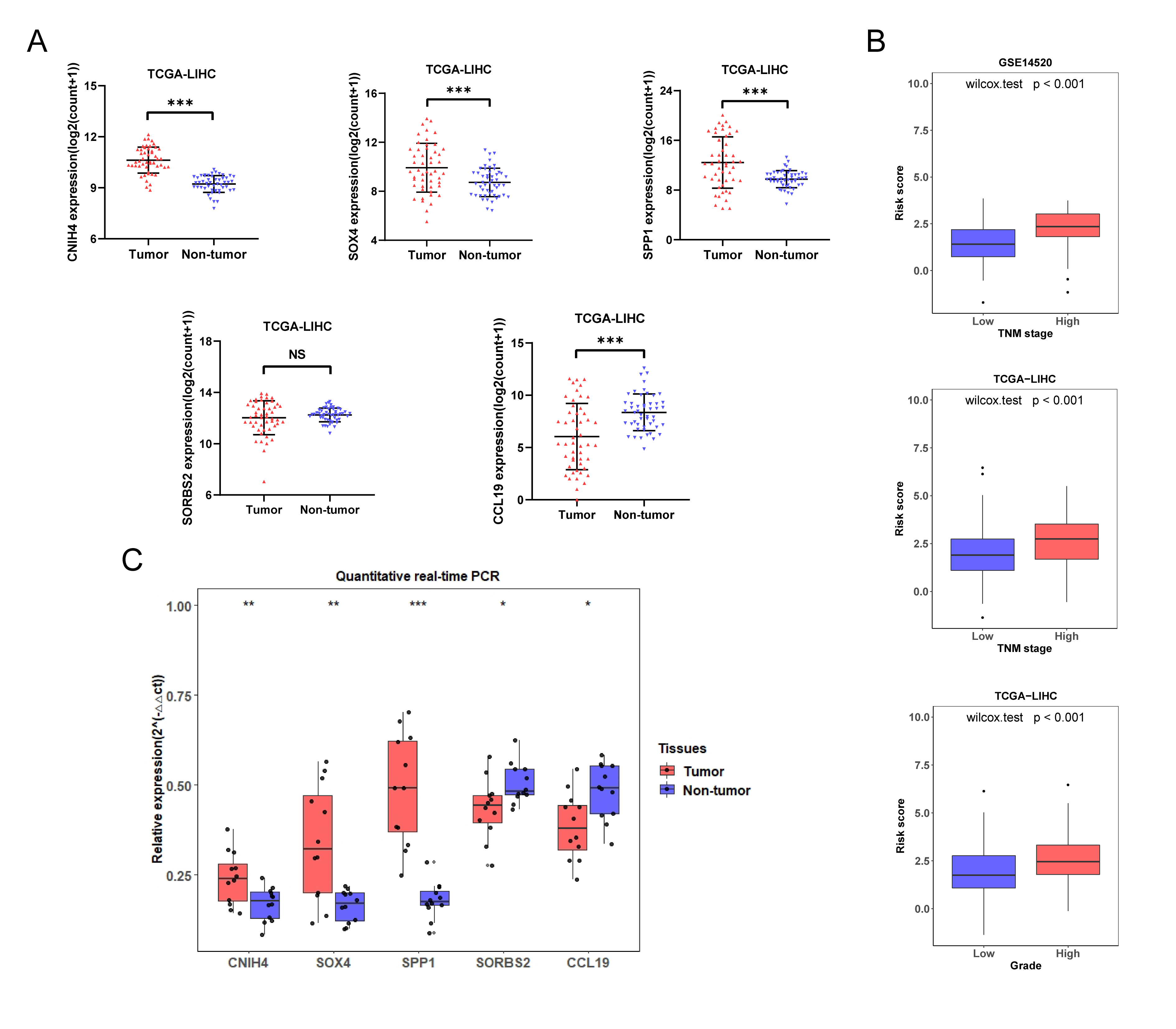

Supplement: Supplementary file 3 — Fig S3 [file CAM4-10-3808-s007.tif]
