# Supplementary material for: A novel five‐gene signature predicts overall survival of patients with hepatocellular carcinoma
Source: Cancer Med. 2021 May 2;10(11):3808–21. doi: 10.1002/cam4.3900 (PMC8178492; doi:10.1002/cam4.3900)
Supplement: Supplementary file 4 — Table S1 [file CAM4-10-3808-s003.docx]

The primer sequences of quantitative real-time PCR.

| Gene symbol | Primer sequence | Tm |
| --- | --- | --- |
| *CNIH4* | F: TCAACTTACCTGTTGCCACTTG | 60.7 |
|  | R: TCTGTTGGATCAAACACTCCCA | 61.2 |
| *SOX4* | F: GACCTGCTCGACCTGAACC | 62.0 |
|  | R: CCGGGCTCGAAGTTAAAATCC | 61.0 |
| *SPP1* | F: CTCCATTGACTCGAACGACTC | 60.2 |
|  | R: CAGGTCTGCGAAACTTCTTAGAT | 60.0 |
| *SORBS2* | F: ACAACCCACCCTACAGTGCT | 63.0 |
|  | R: GGACGCATCCTTAAAGGCATTG | 61.8 |
| *CCL19* | F: TACATCGTGAGGAACTTCCACT | 60.5 |
|  | R: CTGGATGATGCGTTCTACCCA | 61.6 |
| *GAPDH* | F: GGTATCGTGGAAGGACTCAT | 60.2 |
|  | R: CCTTGCCCACAGCCTTG | 60.7 |

The clinicopathological characteristics of patients for qPRC.

| Number | Gender | Age | BMI | TNM |
| --- | --- | --- | --- | --- |
| patient1 | Male | 54 | 21.5 | Low |
| patient2 | Female | 61 | 22.1 | High |
| patient3 | Male | 65 | 19.5 | High |
| patient4 | Male | 47 | 23.5 | High |
| patient5 | Male | 58 | 20.4 | Low |
| patient6 | Female | 72 | 19.5 | Low |
| patient7 | Female | 67 | 19.8 | Low |
| patient8 | Male | 55 | 20.8 | Low |
| patient9 | Male | 59 | 19.7 | High |
| patient10 | Female | 70 | 22.3 | High |
| patient11 | Male | 72 | 19.2 | High |
| patient12 | Female | 68 | 21.1 | Low |
